# Research Progress of Biological Feed in Beef Cattle

**DOI:** 10.3390/ani13162662

**Published:** 2023-08-18

**Authors:** Longteng Ma, Lifen Wang, Zixi Zhang, Dingfu Xiao

**Affiliations:** Animal Nutritional Genome and Germplasm Innovation Research Center, College of Animal Science and Technology, Hunan Agricultural University, Changsha 410128, China; malongteng2021@163.com (L.M.); wanglifen1118@163.com (L.W.); zhangzixi0305@163.com (Z.Z.)

**Keywords:** biological feed, cattle, immune function, enzyme, fermentation

## Abstract

**Simple Summary:**

Developing new, ecologically healthy feed has always been the focus of sustainable animal husbandry development and has been a research hotspot in academic circles. Currently, the bio-feed industry is developing rapidly and efficiently. The resources of feed raw materials are constantly being enriched and expanded, strain screening research is gradually deepening, production technologies such as enzyme preparations and bacterial preparations are continuously being optimized and upgraded, and the number of professional livestock personnel is constantly increasing. The development of bio-feed has broad application prospects. However, there are increasing problems facing biological feed. Therefore, this paper summarizes the classification, function, application, and existing issues of biological feed in livestock and poultry production to provide a reference for subsequent related research and applications.

**Abstract:**

Biological feed is a feed product developed through bioengineering technologies such as fermentation engineering, enzyme engineering, protein engineering, and genetic engineering. It possesses functional characteristics of high nutritional value and good palatability that can improve feed utilization, replace antibiotics, enhance the health level of livestock and poultry, improve the quality of livestock products, and promote a better breeding environment. A comprehensive review is provided on the types of biological feed, their mechanism of action, fermenting strains, fermenting raw material resources, and their current status in animal production to facilitate in-depth research and development of applications.

## 1. Introduction

Developing new, ecologically healthy feed has always been the focus of sustainable animal husbandry development, and has been a research hotspot in academic circles [1]. Biological feed research began in the 1980s but truly developed in the 1990s. Research on bioactive feed additives has made rapid progress, particularly in physiological and biochemical studies of these additives. Currently, biological feed has shown a rapid and efficient trend in both research and practical application. Biological feed mainly includes feed enzyme preparations, feed amino acids and vitamins, prebiotics (direct feeding of microorganisms), oligosaccharides for feed, natural plant extracts, bioactive oligopeptides, etc. [2,3,4,5,6]. During the biological treatment process, the crude fiber is broken down into small molecular substances such as monosaccharides, disaccharides, amino acids, and other small molecules that are easily digestible and absorbable by animals. This improves the feed’s digestion and absorption rate [4]. It also produces and accumulates a large number of nutrient-rich microbial body proteins, as well as useful metabolites such as organic acids, alcohols, aldehydes, esters, vitamins, antibiotics and trace elements. These components make the feed soft and fragrant while increasing its nutritional value. Additionally, they contain various digestive enzymes and unknown growth factors that enhance animals’ disease resistance and stimulate their growth and development [5].

Probiotics are bacteria that help maintain the natural balance of microorganisms in the gut and have beneficial health effects for humans and animals. Studies on non-ruminant animals have shown that the probiotic *Saccharomyces cerevisiae* boulardii (SCB) can reduce diarrhea and promote growth rates in pig and broiler production [3,4,6]. Conflicting findings have been reported regarding the impact of *S. cerevisiae* on health and performance in young calves [7]. Supplementation of SCB did not show any overall effects on the performance, health, or fecal microbial groups in healthy calves [8]. However, SCB supplementation may have a potential antiviral effect that could enhance animal performance for calves with inadequate passive transfer [9]. Similar results have also been reported in young calves using other probiotics [10]. For example, Oikonomou et al. [11] found that calves with neonatal diarrhea and pneumonia had lower bacterial diversity in their feces compared to healthy calves. One of the mechanisms through which SCB improves gut health, as demonstrated in monogastric experimental models, is by enhancing microbial diversity [12,13]. Since neonatal and preweaning dairy calves function as monogastrics, probiotics such as SCB show strong promise.

Beef has the advantages of high protein, low fat, more phospholipids, less cholesterol, delicate and soft muscle fibers, and a unique flavor. With the improvement of people’s living standards and changes in dietary habits, beef consumption has continued to rise steadily, presenting exceptional opportunities for the development of beef production. This paper provides a comprehensive review of the various types, mechanisms of action, and fermentation cultures utilized in bio-fermented feed. The utilization status of fermentation raw material resources in animal production is discussed, which offers a scientific basis for enhancing beef cattle meat quality and practical guidance for beef cattle production.

## 2. Classification and Function of Biological Feed

### 2.1. Classification of Biological Feed

The standard of “Biological Feed Product Classification” categorizes biological feed products into fermented feed, enzymatic hydrolysate feed, bacterial enzyme synergistic feed and biological feed additives. Based on distinct characteristics and production processes, the biological feeds are further classified into 4 main categories, 10 subcategories, 17 sub-subcategories, 50 sub-sub-subcategories, and 112 product categories [1].

#### 2.1.1. Fermented Feed

**Figure 1:**
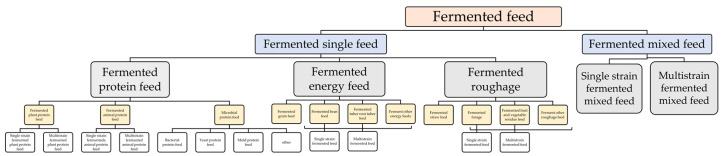
Classification diagram of fermented feed products.

According to the characteristics of the strain, it is mainly divided into oxygen consumption fermentation, anaerobic fermentation, and facultative anaerobic fermentation. The classification is illustrated below in Figure 1. According to the number of strains, it is mainly divided into single-strain fermentation, multi-strain fermentation, bacterial enzyme collaborative fermentation, and other types. The production of feed enzyme preparations is mainly based on the liquid deep fermentation of single strains, while the production of fermented feed raw materials and mixed feed mainly involves the fermentation of compound strains. The selection of fermented feed raw materials has evolved from fermenting soybean meal, cottonseed meal, and rapeseed meal to provide high-quality protein feed. It now focuses on fermenting unconventional feed raw materials such as fresh residue, pomace, and stale vegetables to provide high-quality and low-cost fermented energy feed and roughage.

#### 2.1.2. Enzymatic Hydrolysis Feed

**Figure 2:**
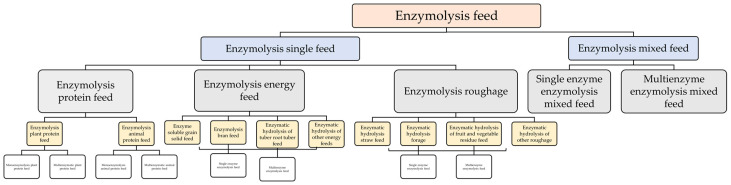
Classification diagram of products from enzymatic hydrolysis feed.

Enzymatic hydrolysis feed has been extensively used in livestock and poultry breeding, with the process parameters and treatment efficacy of pre-digestion of enzymatic feed having been duly validated. The levels of inorganic phosphorus, acid detergent, and reducing sugar serve as crucial indicators that reflect the pre-digestion efficacy of enzyme-based feed in vitro [14]. Optimizing the reaction conditions for feed enzymes can significantly enhance enzymatic pre-digestion of feed under in vitro conditions, leading to improved feed utilization, reduced formulation costs, and enhanced animal performance [14,15]. The classification is illustrated in the Figure 2 below.

#### 2.1.3. Cooperation of Bacteria and Enzyme

**Figure 3:**
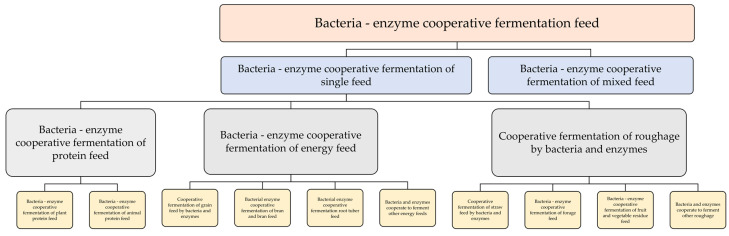
Classification diagram illustrating the symbiotic relationship between bacteria and enzyme products.

In recent years, research findings have demonstrated that the co-treatment of bacterial enzymes yields superior results compared to the individual actions of bacteria and enzymes; the classification is illustrated below in Figure 3. During the process of fermentation, microorganisms and enzymes work in remarkable synergy, resulting in a more thorough degradation of macromolecular substances and enhancing the efficiency of microbial fermentation. The synergistic effect of microorganisms and enzymes is utilized to gradually increase the preparation of relevant reports on fermented feed [15,16,17,18,19]. The synergy of bacterial enzymes can not only shorten the fermentation cycle, but also utilize bacillus or lactic acid bacteria to resist the influence of other bacteria, improve efficiency, reduce production costs and alter the microecological environment in animal intestines through a large number of live bacteria such as bacillus, lactic acid bacteria, and yeast contained in the product. This enhances animals’ resistance to diseases and reduces their reliance on antibiotics [20,21,22,23].

#### 2.1.4. Biological Feed Additives

**Figure 4:**
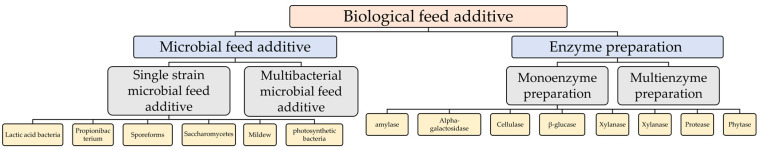
Classification diagram of products for biological feed additives.

Bioengineering technology has been applied to develop a range of feed additives that can enhance feed utilization efficiency, improve animal health, and boost production performance. These additives include microbial feed additives, enzyme preparations, and oligosaccharides [14,15]. The classification is illustrated below in Figure 4.

### 2.2. Biological Feed Function

Biological feed can enhance palatability, reduce anti-nutritional factors, optimize nutrient utilization, facilitate the efficient use of agricultural and agricultural by-products, minimize reliance on pharmaceutical additives such as antibiotics, and ultimately improve livestock and poultry production performance [24,25,26]. There are various types of raw materials for feed production in China, and the selection criteria for biological feed should be based on differences in physical and chemical properties of the raw materials, physiological characteristics of strains, and production purposes.

#### 2.2.1. Improve the Immune Function of Livestock and Poultry

It is found that after feed fermentation, the number of beneficial microorganisms in feed can be effectively increased so that more beneficial microorganisms can enter the intestines of livestock and poultry to form a competitive advantage and quickly colonize and become dominant flora. For instance, lactic acid bacteria can effectively lower the intestinal pH by producing significant amounts of lactic acid and acetic acid. This promotes the growth of intestinal villi, expands the absorption area of the small intestine, and strengthens the intestinal barrier through competitive inhibition and production of adhesion substances. Additionally, it restricts the adhesion and proliferation of pathogenic bacteria on intestinal mucosal epithelial cells, thereby reducing harmful bacterial populations in the intestines and maintaining a balanced microbial environment, as well as decreasing the incidence of diarrhea in livestock and poultry [27,28]. As non-specific immunomodulators, probiotics and metabolic active substances in fermented feed can stimulate the development of intestinal immune organs in livestock and poultry, activate the immune mechanism of the intestine, improve the cellular immunity and humoral immunity of livestock and poultry bodies, increase the content of immunoglobulins in the blood of livestock and poultry, and promptly eliminate them to eradicate pathogenic bacteria that invade the animal, improve the resistance of livestock and poultry to diseases, and reduce the occurrence of diseases [29,30,31,32,33]. Previous research from the preceding period has also demonstrated that certain probiotics exhibit efficacy as immunomodulators by modifying the immune response of T-helper cells 1 and 2, thereby augmenting host immune function [34].

#### 2.2.2. Enhance the Gastrointestinal Microecological Environment and Production Environment of Livestock and Poultry

Although the effect of probiotics on gut microbial composition has been extensively studied in swine and poultry [35], it is still largely unknown how probiotics potentially affect the gut microbiota in calves. Fomenky et al. [36] observed significant changes in gut mucosa/digesta microbial composition in calves supplemented with SCB before and after weaning. Therefore, intestinal mucosa samples should be collected in future studies with a higher number of animals because mucosa-associated bacteria interact more closely with the host gut health [37]. The potential modulation of host immune function, which can be mediated by regulating the gut microbiota, could be consequently highlighted. However, it should be noted that only four calves per treatment were used for microbial analysis in their experimental study and the small sample size might overestimate the treatment effect [38].

*Fecalibacterium* is recognized as a prominent butyrate producer, and the presence of butyrate has been shown to potentially enhance the integrity of the intestinal epithelial barrier [39]. A greater prevalence of *Fecalibacterium* spp. In fecal specimens from neonatal calves was linked to increased weight gain and reduced incidence of diarrhea in preweaning dairy calves [11], while the occurrence of *Fecalibacterium* spp. Was observed to be reduced in canines suffering from acute diarrhea [40]. On the contrary, in vitro studies have shown that Collinsella can decrease the expression of tight junction proteins in intestinal epithelial cells, leading to an increase in gut permeability. Additionally, it has been observed to upregulate the production of pro-inflammatory factors such as IL-17, both of which contribute to the disruption of gut epithelial integrity [41]. *Lactobacillus* has been demonstrated to reduce the incidence of diarrhea in calves [42], while Escherichia-Shigella comprises several opportunistic pathogens, such as enteropathogenic *E. coli* and Shiga toxin-producing *E. coli* [43], which can cause diarrhea in calves. An increase in the abundance of *Lactobacillus* and a decrease in *E. coli* could help ensure normal growth performance of calves even when they experience diarrhea [44].

#### 2.2.3. Improve Feed Utilization and Growth Performance

After fermentation, feed can produce a variety of unsaturated fatty acids and aromatic compounds that impart unique flavors and enhance palatability. This leads to a significant increase in feed intake among livestock and poultry. Additionally, biological feed generates proteases, amylases, cellulases, phytases, water-soluble vitamins, amino acids, and various growth-promoting factors upon metabolism within the digestive system of animals. These components promote optimal growth and development in livestock and poultry [45,46,47]. Moreover, bio-fermented feed can establish a dense microbial barrier in the intestines of livestock and poultry, which effectively prevents the absorption of harmful substances and waste [31,46,47,48,49]. The incorporation of probiotic fermented feed into the diets of dairy cows has been shown to enhance milk production, improve apparent nutrient digestibility in lactating cows, and elevate milk quality [50].

## 3. Biological Feed Strains and Enzyme Preparations

### 3.1. Classification and Characteristics of Strains

#### 3.1.1. Yeast

Yeast is widely distributed in nature, with a fast reproduction rate, short growth cycle, and strong metabolism. This includes tropical candida, prion-producing candida, brewer’s yeast, red yeast, among others. Yeast is a rich source of B vitamins, proteins, amino acids and other nutrients that can effectively enhance the nutritional value of animal feed, improve its digestibility and utilization by livestock and poultry, boost their immune system function and suppress the growth of harmful microorganisms [51,52,53]. The utilization of live yeast *S. cerevisiae* as a probiotic supplement in ruminant feed has been shown to enhance the growth performance and feed efficiency of fattening calves [54]. Villot et al. [55] demonstrated the effects of *Saccharomyces cerevisiae* (SCB) on the growth performance, health status, and fecal microorganisms of Holstein calves aged 6 ± 3 days. However, supplementation with yeast (10 × 10^9^ cfu/d) did not improve the average daily gain, final weight, dry matter intake (DMI), and other performance indicators. On a positive note, it did decrease the incidence of diarrhea in calves. Lee et al. [56] demonstrated that the inclusion of SCB (10 × 10^9^ cfu/d) in the diet resulted in a significant increase in DMI and water intake under thermoneutral conditions, while concurrently reducing the incidence of diarrhea. Crossland et al. [57,58] also discovered that the inclusion of dry yeast in the diet contributed to the maintenance of rumen environment stability.

#### 3.1.2. Lactic Acid Bacteria

In the field of biological feed, the majority of previous research has primarily focused on exploring the application of diverse strains of lactic acid bacteria [20,59]. Lactic acid bacteria, including homotypic lactic acid bacteria, heterotypic lactic acid bacteria, facultative lactic acid bacteria, and bifidobacteria, are the predominant probiotics utilized in silage preservation due to their early discovery and extensive application. Lactic acid bacteria can enhance feed palatability, stimulate the secretion of digestive enzymes, organic acids, vitamins and other bioactive substances in the gastrointestinal tract of livestock and poultry, promote colonization by beneficial bacteria, and inhibit the proliferation of spoilage microorganisms to maintain a balanced intestinal microecology [60], as well as improve the effectiveness of animal feed utilization and maximize the overall growth performance of animals [54].

#### 3.1.3. Mold

Mold is a broad term encompassing filamentous fungi, and mold fermentation yields functional enzyme fungi with a wider range of enzymes than bacteria, including cellulase, protease, phytase, among others. Approximately 90% of the enzyme preparations listed in the Feed Additives Catalogue are derived from mold sources. However, mycotoxins produced by molds pose serious threats to feed quality and livestock safety; examples include zearalenone, vomitoxin, aflatoxin B1 [29,61,62].

#### 3.1.4. Bacillus

*Bacillus*, including *Bacillus cereus*, *Bacillus subtilis*, *Bacillus macrosporidus*, and *Bacillus licheniformis*, is present in the intestines of animals. It can tolerate gastric acid and has strong resistance to feed processing engineering. Additionally, it exhibits high stability and possesses high levels of protease, amylase, and lipase activities. Furthermore, it can degrade some complex compounds found in plant feed [63,64]. *Bacillus* strains, such as *Bacillus licheniformis* and *Bacillus subtilis*, are commonly used in the feed industry due to their ability to effectively increase soluble protein content while reducing sugar levels, amino acid concentrations, and cellulose degradation rates [65].

*Bacillus* species are not typically indigenous to the gastrointestinal tract. Nevertheless, it has been reported that the oral administration of adequate amounts of *Bacillus subtilis* can favorably modulate the microflora balance in the gastrointestinal tract and enhance animal performance [25,66]. Moreover, numerous publications have suggested that *B. subtilis* possesses remarkable immunostimulatory properties [67] and may effectively facilitate the secretion of certain beneficial vitamins, such as vitamin K [34,68]. Various probiotic supplements can also shorten the duration of bovine diarrhea, albeit without any impact on daily weight gain [69]. Khaziahmetov et al. [70] also acknowledged the remarkable potential of “Stimix Zoostim” probiotics in reducing feed costs, enhancing average daily gain and protein digestibility, as well as elevating erythrocyte, hemoglobin, and gammaglobulin levels. Mousa et al. [71] demonstrated that the inclusion of *Bacillus subtilis* in the diet of calves resulted in increased dry matter intake, digestibility, final weight gain, and feed conversion efficiency. Additionally, there was an increase in total volatile fatty acids and ammonia nitrogen concentration within the rumen while protozoal populations were reduced. Blood levels of total protein, albumin, triglycerides, aspartate aminotransferase and glutamic-pyruvic aminotransferase were also significantly elevated along with lower levels of urea and creatinine.

### 3.2. Enzyme Preparations

The enzyme preparations belong to a group of proteins possessing biocatalytic capabilities that can be derived from animals, plants, and microorganisms. They include single enzyme preparations (such as amylase, phytase, protease, cellulase) and complex enzyme preparations (which are mixtures of multiple single enzyme preparations) [72]. Enzyme preparations possess the characteristics of being environmentally friendly, safe, and efficient. They can effectively break down complex structural substances in feed, improve protein content, soluble carbohydrate content, and other nutrients to enhance feed utilization. Additionally, they promote intestinal microflora balance, endocrine regulation, and metabolism while also facilitating nutrient absorption [73]. Meschiatti et al. [2] discovered that the addition of exogenous α-amylase has a synergistic effect with essential oils and their blends, leading to enhanced performance and ketone weight in beef cattle when compared to Monensin. The research conducted by Vigne et al. [74] on fattening animals suggests that the inclusion of enzyme complexes in feed can improve the feed conversion rate by 0.17% per gram, reduce stool dry matter content by 0.47% on the first day, and decrease drinking time by 0.0068 h. Marwan et al. [75] investigated the impact of exogenous plasminase on the digestibility and performance of buffalo calves. The inclusion of enzyme preparation significantly enhanced DMI, nutrient digestibility, average daily gain (ADG), final weight, and feed conversion rate in the calves. Additionally, there was a notable increase in rumen total volatile fatty acid (TVFA), ammonia concentration, protozoan number, total protein (TP), and albumin (ALB) levels. However, no significant effects were observed on urea levels or indicators such as muscle crispness, triglyceride content, aspartate aminotransferase (AST) and alanine transaminase (ALT) enzymes. In their study on *Bacillus subtilis* (DSM 28343), Bampidis et al. [76] concluded that the addition of this bacterium to calf feed had a positive impact on the structure of intestinal flora.

## 4. Application of Biological Feed in Cattle Breeding

The application of biological feed in ruminants primarily helps the rumen establish a dominant flora, regulates rumen function and microflora, promotes rumen metabolism, increases antioxidant capacity and immunity [57,58], and subsequently promotes ruminant growth and improves production performance [71].

Stressful environmental factors, such as warmer temperatures and high humidity, can cause heat stress in calves. Exposure to heat stress results in poor growth performance due to reduced feed intake [77,78], impaired homeostatic mechanisms, and altered physiological status of the endocrine and immune systems [77,79,80]. Reduced feed intake is a major concern for farmers and livestock owners because it can lead to a host of negative consequences. When coupled with lower physiological responses, such as decreased metabolic rate and weakened immune system, the effects can be even more devastating.

One of the most common outcomes of reduced feed intake and lowered physiological responses in calves is poor growth. Without adequate nutrition, young animals are unable to develop properly and may suffer from stunted growth or malnourishment.

Another serious consequence is the outbreak of calf diarrhea. This condition can be caused by a variety of factors, including changes in diet or stress on the animal’s digestive system. However, when combined with reduced feed intake and weakened immunity, it becomes much more likely to occur—leading to dehydration, weight loss, and potentially fatal complications.

In extreme cases where these conditions go untreated or unaddressed for too long, death may unfortunately become an outcome. It is crucial that farmers take steps to ensure their livestock receive proper nutrition and care at all times in order to prevent these negative outcomes from occurring.

By supplementing 0.5 g of a product containing *Saccharomyces cerevisiae* CNCM I-1077 (2 × 10^10^ cfu/g) to grain, the intake and growth rate of immature animals can be significantly enhanced prior to weaning [9]. However, the incorporation of 1.0 g of a product containing *Saccharomyces boulardii* (SB) CNCM I-1079 (2 × 10^10^ cfu/g) into milk replacer fails to enhance the dry matter intake or promote growth in immature calves [81]. Intriguingly, calves experience reduced incidences of diarrhea or pneumonia when administered yeasts, irrespective of dosages or strains [9,81], without considering the influence of ambient temperature and humidity. Calves experiencing heat stress exhibit an elevated body temperature, which may lead to increased intestinal permeability of enteric Pathogens [82,83,84]. Calves exposed to lipopolysaccharide (*E. coli* O55:B5) exhibit an elevation in body temperature [85]. Meanwhile, yeast has been shown to mitigate the impact of heat stress on undesirable enteric microbial populations [85] and acute inflammatory responses in swine [86]. However, insufficient data is available on calf response to SB coupled to thermal stress in controlled environments.

Previous studies have demonstrated that the supplementation of SCB did not result in any growth or performance improvement in calves, both during the pre-weaning period [8] and post-weaning [36]. In Villot et al.’s research [55], the mortality rate (9.5%) was higher than previously reported in Ontario veal farms [87]. The occurrence of early mortality was significantly correlated with factors such as weight, season, barn, and supplier; late mortality showed significant associations with season of arrival, barn conditions, and supplier [87]. It has been established that early mortality is linked to a failure in passive transfer [88]. When Villot et al. analyzed the data by comparing calves who experienced diarrhea with those who did not within each dietary treatment, they discovered that the diarrheic calves supplemented with SCB maintained similar ADG, matter intake, and final weight compared to their non-diarrheic counterparts. In contrast, the control diarrheic calves had significantly lower growth parameters than the control non-diarrheic calves [55]. Clinical trials have reported reduced weight gain in sick calves treated with antibiotics compared to healthy ones [89].

Roughage, though containing relatively minor nutrients other than neutral detergent fiber and being poorly palatable, is an indispensable component of ruminant feed. By means of microbial fermentation technology, the nutritional characteristics of roughage can be effectively improved [90], and can improve animal production performance [91]. Silage serves as a vital source of energy, nutrients, and easily digestible fiber for ruminants. Incorporating an appropriate amount of corn silage into cow roughage can significantly enhance feed intake, milk production, and milk protein content [9,90,92,93,94]. Biological feed has the ability to regulate the microflora of ruminant rumen, boost immunity and antioxidant capacity, optimize growth performance, and enhance meat quality; the results are shown in Table 1 [54,56,71,95,96,97,98,99,100,101,102,103,104,105]. Other research has demonstrated that the inclusion of silage in dairy cow diets can enhance their immune function and antioxidant capacity, as well as elevate the levels of polyunsaturated fatty acids present in milk [106].

## 5. Conclusions

Biological feed has the ability to regulate the microflora of ruminant rumen, boost immunity and antioxidant capacity, optimize growth performance, and enhance meat quality.

The swift advancement of biological feed holds immense significance in mitigating the dearth of feed resources. The development of biological feed enhances the nutritional value of fodder, curbs resource wastage and environmental pollution, and facilitates cost-effective breeding with high returns, thereby presenting vast prospects for growth.

## Figures and Tables

**Table 1:** The utilization of biological feed in cattle breeding.

Items	Levels	Ruminants	Main Influences	References
*S. cerevisiae* (SC), spp. Boulardii (SCSB)	0.5 g/d	Holstein calves	Calves on SC had higher grain intake, weight gain pre-weaning, and plasma glucose levels than controls. Feeding live yeast to calves reduced diarrhea days, but antibiotic resistance in fecal *E. coli* was highest in 3-day-old calves and not affected by other treatments except for SCSB, which increased resistance levels compared to controls. Improvements in calf performance with failed passive transfer were observed by adding live yeast only to the grain.	Klibs et al., 2005 [9]
Fermented soybean meal	5%	Holstein calves	Calves fed fermented soybean meal (FSBM) exhibited enhanced feed intake, growth performance, and health during the preweaning period, as well as reduced levels of stress-related serum variables (proinflammatory cytokines, acute-phase proteins [APP], and cortisol) following weaning. The incorporation of FSBM in their diet may prove advantageous in mitigating stress responses experienced by calves during the weaning process.	Kim et al., 2014 [95]
Cassava chips or fermented cassava starch residue (FCSR)	100%	Thai native x Lowline Angus crossbred steers	The substitution of FCSR for cassava chips in the concentrate resulted in a decrease in feed digestibility; however, it had no detrimental effects on growth performance and carcass characteristics.	Pilajun et al., 2016 [96]
SmartCare (SC), *Saccharomyces cerevisiae* (XPC)	1 g/d SC; 1 g/d SC + 0.5 g/d XPC	Holstein calves	Supplementing pre-weaned Holstein calves with both SC in milk replacer and XPC in calf starter not only improved starter intake, but also enhanced fecal consistency immediately after a mild Salmonella enterica challenge. However, further data are required to fully comprehend the impact of these yeast fermentation products on immune responses to Salmonella enterica.	Harris et al., 2017 [97]
*Saccharomyces cerevisiae* var boulardii (SCB)	10 × 10^9^ cfu/d	Holstein calves	Supplementation of SCB did not yield any discernible effects on the performance, health, or fecal microbial groups in healthy calves.	He et al., 2017 [8]
Fermented fiber (FFS)		Holstein bull calves	The findings revealed that despite the reduction in feed intake and increased water content in feces, the high starch diet exhibited a remarkable enhancement in the energy status of the calves by reducing BHB levels, elevating propionate concentration, and optimizing glucose concentrations. Notably, when compared to FFS, the SS-based diet demonstrated superior efficiency in bull calves.	Kazemi-Bonchenari et al., 2017 [98]
SB-300 (Croton lechleri standardized botanical extract)	1000 mg/d	Holstein calves	The inclusion of 500 mg SB-300 in milk for a duration of 15 days can effectively mitigate the incidence of diarrhea and alleviate severe dehydration in calves that are fed with milk.	Teixeira et al., 2017 [99]
Grape pomace	10%	Cattle	Reflects antioxidant activity.	Iannaccone et al., 2018 [100]
*Saccharomyces cerevisiae* boulardii CNCM I-1079 (SCB)	10 × 10^9^ cfu/d	Holstein calves	SCB has beneficial effects on health parameters of milk-fed calves raised in a commercial veal farm environment.	Villot et al., 2019 [55]
*Saccharomyces boulardii* CNCM I-1079 (SB)	10 × 10^9^ cfu/d	Holstein calves	Supplementation of SB in milk replacer can mitigate the effects of heat stress on Holstein dairy calves, as evidenced by lowered rectal temperature, heart rate, and potential incidence of diarrhea.	Lee et al., 2019 [56]
10% dry (AH) or reconstituted alfalfa hay (RAH)	91.2 and 83.8% dry matter for AH and RAH, respectively	Holstein dairy calves	Reconstitution of alfalfa hay did not have an impact on dry matter intake, growth performance, and metabolic indications of rumen development during the calf phase. However, it did lead to improvement.	Kargar et al., 2019 [101]
*B. subtilis*	0.3 gm/1 kg	Buffalo calves	The findings of this study suggest that *B. subtilis* has the potential to serve as a beneficial probiotic for buffalo calves, enhancing their dry matter intake, digestibility, average weight gain, and feed conversion ratio when administered at an effective daily dose of 0.3 gm/1 kg in their diet.	Sa et al., 2019 [71]
Fermented corn gluten meal (FCGM)	5%	Holstein male calves	The inclusion of FCGM in the diet resulted in increased growth rate and feed efficiency, enhanced rumen fermentation, diversified bacterial community composition within the rumen, as well as improved antioxidant and immune functions of postweaning calves.	Jiang et al., 2019 [102]
Fermented corn gluten meal (FCGM)	6%	Holstein calves	The findings suggest that pre-weaning feeding of FCGM in calves resulted in enhanced growth performance, reduced incidence of diarrhea, increased antioxidant and immune capacity, improved rumen function, as well as altered bacterial community composition in both rumen fluids and feces.	Jiang et al., 2020 [103]
*Bacillus* Megaterium (BM)	1259 powder (1 × 10^10^ cfu/g)	Holstein bull calves	This study demonstrates that BM1259 has the potential to serve as a microecological agent for enhancing growth performance, nutrient digestibility, rumen fermentation, and nitrogen utilization in Holstein bull calves.	Deng et al., 2021 [104]
Live yeast *S. cerevisiae*	28 g/d	Holstein calves	This supplementation significantly improves the mean daily gain during the trial (ADG) by 400 g/calf (*p* < 0.003). A notable increase in final body weight gain (FWG) of 39.1 kg/calf was observed (*p* < 0.004). The live yeast supplementation reduces feed intake and significantly lowers the average feed conversion rate (FCR) (*p* < 0.05). The use of *S. cerevisiae* as a probiotic supplement in ruminant feed enhances growth performance and feed efficiency in fattening calves.	Maamouri et al., 2021 [54]
*Clostridium butyricum* (CB)	2 × 10^8^ cfu	Holstein heifers	Supplementation with CB resulted in a significant decrease in the proportion of acetate and the ratio of acetate to propionate, while increasing the proportion of butyrate and propionate. Compared to the control group, the CB group had higher levels of *Butyrivibrio fibrisolvens*, *Ruminococcus albus*, *Ruminobacter amylophilus*, *Ruminococcus flavefaciens*, and *Streptococcus bovis* during the trial period. However, CB failed to elicit a significant impact on blood parameters in Holstein heifers. In conclusion, these findings evince that the administration of CB can augment growth performance and rumen fermentation without any deleterious effects on blood parameters in Holstein heifers.	Yang et al., 2021 [105]

## Data Availability

Data openly available in a public repository.
